# Enhancing hydrogen uptake in TiFe at moderate pressures *via* rationally designed biomass-derived carbon additives

**DOI:** 10.1039/d5ra07863f

**Published:** 2026-01-02

**Authors:** Madina Kalibek, Nurbolat Issatayev, Aigerim Ospanova, Talgat Orazbek, Ayaulym Amankeldiyeva, Mirat Karibayev, Vladislav Kudryashov, Aitkazy Kaisha, Dhawal Shah, Nurxat Nuraje

**Affiliations:** a Renewable Energy Lab, National Laboratory Astana, Nazarbayev University Astana 010000 Kazakhstan nurxat.nuraje@nu.edu.kz; b Department of Chemical and Materials Engineering, School of Engineering and Digital Science, Nazarbayev University Kabanbay Batyr Ave. 53 Astana 010000 Kazakhstan

## Abstract

Hydrogen is a promising clean energy carrier, but its widespread use is limited by challenges in safe, efficient, and scalable storage. TiFe alloy is an attractive solid-state hydrogen storage material due to its high capacity and operation under mild conditions, yet its practical performance is hindered by surface oxidation, which impairs hydrogen sorption uptake. A sustainable approach uses activated carbon made from agricultural waste to overcome this limitation. By screening activated carbons produced from three biomass precursors, we identify garlic-peel-derived carbon as an optimal additive, exhibiting an ultrahigh surface area (∼3200 m^2^ g^−1^), oxygen-containing functionalities, a predominantly microporous architecture (83%), and high hydrogen uptake (0.99 wt% H_2_ at 298 K and 100 bar). Incorporation of only 1 wt% of this material into TiFe suppresses oxide formation and increases hydrogen uptake from ∼1.4 to 1.5 wt% under identical conditions. Additionally, molecular dynamics simulations reveal that incorporating a porous activated carbon layer (0.65 nm pores) onto TiFe alloys significantly alters hydrogen distribution by introducing additional sorption sites. This approach improves hydrogen storage and promotes sustainability by converting agricultural waste into a valuable material.

## Abstract 2

Biomass-derived activated carbon suppresses TiFe surface oxidation and enhances hydrogen storage, enabling a sustainable route to improved solid-state hydrogen uptake.

## Graphical abstract

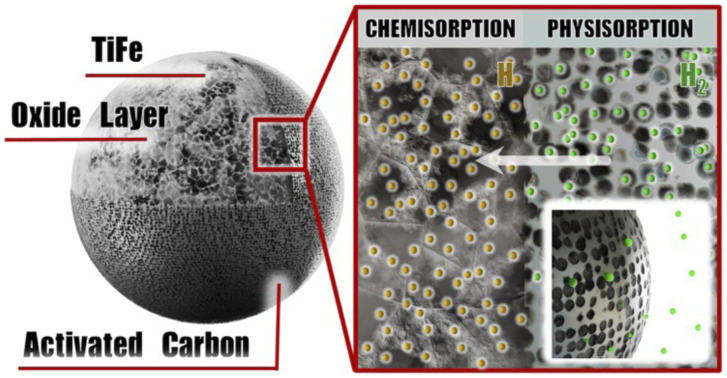

## 1. Introduction

Renewable energy sources have garnered attention due to environmental degradation caused by fossil fuels, positioning their adoption as a key strategy for reducing emissions and achieving climate neutrality. Among these, hydrogen energy stands out as a promising option due to its renewability, environmental compatibility, and high gravimetric energy density.^1^ However, the extensive utilization of hydrogen as a sustainable fuel for transportation is impeded by persistent technological challenges, most notably the lack of practical and high-efficiency storage technologies. As a result, solid-state hydrogen storage has attracted increasing interest due to its higher volumetric capacity, greater safety and convenience compared to conventional gaseous and liquid methods.^2,3^ Key materials for hydrogen storage, particularly in applications such as hydrogen fuel tanks, must exhibit a combination of desirable properties, including the ability to absorb hydrogen at ambient temperature, high hydrogen storage capacity, suitable equilibrium pressure, and ease of activation.^4^ Various materials,^5–8^ such as metal hydrides, porous materials, and composites, have been extensively investigated for this purpose.

Among these materials, TiFe alloy is considered one of the most promising materials for solid-state hydrogen storage due to its relatively high hydrogen capacity, fast kinetics, and use of low-cost, abundant elements.^9–13^ TiFe alloy-based systems can operate under mild conditions and offer a high volumetric hydrogen density, making them attractive for stationary and naval applications.^10,12^ However, activation process of TiFe needs to be improved to suppress surface oxidation, which prevents hydrogen uptake.^12^ Specifically, the decline in absorption capacity is mainly because Ti_10_Fe_7_O_3_ and Ti_3_Fe_3_O cannot react with hydrogen, and the hydride formed from Ti_4_Fe_2_O_*x*_ is unstable, requiring high temperatures or low pressures for hydrogen release.^14,15^ The activation mechanism of TiFe has not been quite well understood. Falcão *et al.* reported that hydrogen absorption activity in the TiFe compound immediately after the milling process, without requiring any additional activation treatment to operate effectively at room temperature.^10^

Alongside TiFe, biomass-derived activated carbons represent a promising class of materials for hydrogen storage due to a high specific surface area, lightweight, chemical stability and fast kinetics.^16^ Biomass serves as an excellent natural carbon precursor due to its abundance, cost-effectiveness, and sustainability.^17^ Numerous biomasses^1,18–22^ have been successfully repurposed for production of carbon materials. Analysis of published studies on biomass-derived activated carbons reveals that their practical application remains constrained by intrinsic limitations, including low carbon yield, underdeveloped pore architectures, and significant hydrogen uptake occurring only at moderate pressures or under cryogenic conditions.

The hydrogen uptake of most studied materials typically falls short of the thresholds required for practical applications. Combining different materials can yield synergistic effects that enhance sorption performance. Undoubtedly, various additives^23,24^ can be used to enhance uptake or kinetics. Notably, it was found that carbon materials can be used as additives for metal hydrides, as they are light, relatively inert, often abundant, and efficient heat conductors, beneficial for heat transport during hydride formation, while also in some cases enhancing hydrogen uptake, increasing plateau pressure, and passivating the surface of highly reactive metals.^7,25–28^ Balcerzak and colleagues also investigated materials catalyzed by carbon additives (SI Fig. S1), and noted the lack of related studies, underscoring a significant gap in the literature.^25^

In this work, we rationally select biomass-derived activated carbon additive for TiFe by examining specific surface area, oxygen-containing surface functionalities, microporous structure, and hydrogen uptake. We further compare the resulting hydrogen uptake of TiFe with that of TiFe containing the additive to elucidate how the activated carbon enhances the hydrogen-storage performance. Particular attention is given to pressures ≥10 bar, where TiFe enters its absorption plateau corresponding to full hydride formation, and the activated carbon contributes additional storage capacity through significant hydrogen adsorption. As discussed earlier, the carbon serves multiple functions: it prevents oxidation, adsorbs hydrogen molecules, and, most importantly, is lightweight and chemically inert. Abundant biomass precursors such as garlic peel, spent coffee grounds, and onion peel were used to synthesize the carbon. The synthesis process of biomass-derived carbon involves straightforward steps and yields a highly porous and chemically pure material. Among all the tested precursors, garlic peel-derived activated carbon resulted in the highest hydrogen uptake: 0.99 wt% H_2_ at 298 K (6.96 wt% H_2_ at 77 K) under 100 bar pressure. To the best of our knowledge, this value is among the highest reported for raw biomass-derived activated materials. Incorporating just 1 wt% of the carbon into the alloy enhances hydrogen uptake from ∼1.4 wt% H_2_ till ∼1.5 wt% H_2_ (298 K and 100 bar), owing to both the inhibition of TiFe oxidation and the carbon's inherent sorption ability, with no indication of chemical bonding between the two components.

## 2. Materials and methods

### 2.1 Materials

Garlic peel, spent coffee grounds and onion peel were used as biomass precursors for carbon synthesis. Potassium hydroxide (KOH; pellet form, ≥85% purity; Sigma-Aldrich) served as the chemical activating agent. Hydrochloric acid (HCl; Sigma-Aldrich) was diluted from ∼12 M (≥37%) to 1 M and used for post-synthesis washing to remove inorganic impurities. High-purity nitrogen gas (N_2_, purity ≥ 99.999%; IhsanTechnogaz) served as the inert atmosphere during carbonization and activation processes.

Titanium powder (Ti, purity 99.98%, particle size < 45 µm; Sigma-Aldrich) and iron powder (Fe, purity 99.74%, particle size < 160 µm; KazMetService) were used as raw materials for alloy preparation. All metallic powders were handled under an inert atmosphere inside an nitrogen-filled glove box (N_2_ purity ≥ 99.99%; IhsanTechnogaz) to avoid oxidation and moisture contamination.

Hydrogen sorption measurements were conducted using high-purity hydrogen gas (H_2_, purity ≥ 99.999%; Topan).

### 2.2 Preparation of biomass-derived activated carbon additives

**Fig. 1:**
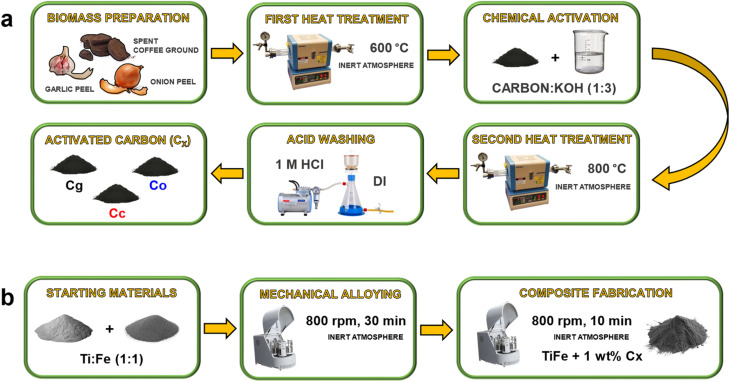
(a) Sequential synthesis methodology of biomass-derived activated carbon additive. (b) Sequential synthesis methodology of TiFe and composite.

As shown in Fig. 1a, the synthesis methodology of the biomass-derived activated carbon additive comprises several steps. The preparation process begins with thorough washing of the biomass in deionized water, followed by drying at 80 °C and subsequent milling. The initial thermal treatment involves heating the sample to 600 °C in a tube furnace under a nitrogen atmosphere (10 °C min^−1^, 2 h), with continuous temperature monitoring and an intermediate hold at 300 °C for 1 h to facilitate the decomposition of organic constituents. This process yields a solid carbonaceous material (char). Chemical activation is further performed by impregnating the carbon with KOH at a mass ratio of 1 : 3 (carbon : KOH). The resulting mixture was left under a fume hood for 24 h and then heated to 95 °C at a rate of 1 °C min^−1^ until the water evaporated, using a muffle furnace. A second heat treatment is then conducted at 800 °C under nitrogen (10 °C min^−1^, 2 h) using a ceramic tube. The ceramic tube is selected for its high thermal and chemical stability, providing structural integrity under harsh processing conditions. Post-activation, the material is subjected to acid washing with 1 M HCl for 24 h, followed by filtration with deionized water until a neutral pH is achieved. The final drying step is carried out at 80 °C.^29^ The biomass-derived activated carbon additives (C*x*) were defined as Cg (garlic peel-derived activated carbon), Cc (spent coffee ground-derived activated carbon), Co (onion peel-derived activated carbon).

### 2.3 Preparation of TiFe alloy and composite

As shown in Fig. 1b, high-purity Ti and Fe powders were used as starting materials for the mechanical alloying synthesis (referred to as MA synthesis) of TiFe (1 : 1). The mass ratio of Ti to Fe was approximately 0.857 : 1, corresponding to 0.924 g of Ti and 1.076 g of Fe for a total of 2 g of TiFe powder. The powders were loaded into stainless steel jars under an inert atmosphere inside a glove box to prevent oxidation. MA synthesis was performed for 30 min at a rotational speed of 800 rpm.^30^ Stainless steel vials and milling balls (5 mm diameter) were employed, with a ball-to-powder mass ratio of 8 : 1. Following synthesis, 1 g of the resulting TiFe powder was used as the pristine sample for further analysis, while the remaining 1 g was employed for composite preparation. To fabricate the composite, 1 wt% of biomass-derived activated carbon additive was mixed with TiFe and ball-milled at 800 rpm for 10 min (1 wt% is among commonly used amount for effective dispersion,^25^ while 10 min milling minimizes the risk of carbon structure degradation^7,25^). The composite material was defined as TiFe@C*x*, where *x* is the selected biomass-derived activated carbon additive.

### 2.4 Material characterization

Sample morphology was imaged using scanning electron microscopy (SEM, Crossbeam 540, Carl Zeiss). Surface elemental composition was determined by energy-dispersive X-ray spectroscopy (EDS, X-max, Oxford). Surface chemical states were analyzed by X-ray photoelectron spectroscopy (XPS, NEXSA XPS, Thermo Scientific). Bulk elemental composition was measured by inductively coupled plasma atomic emission spectroscopy (ICP, iCap Duo 6300, Thermo Fischer), and CHN elemental analysis (UNICUBE® trace organic elemental analyzer, Elementar) was conducted primarily to quantify the carbon content. Crystalline structures were examined using X-ray diffraction (XRD, SmartLab, Rigaku). Raman spectroscopy (LabRAM HR Evolution & Omega Scope, HORIBA & Aist-NT) was used to assess the degree of graphitization and structural disorder in the carbon materials. Gas Pycnometer (AccuPyc III 1350) was used to measure the true density of carbon powders. Textural properties, including specific surface area, were determined by N_2_ adsorption–desorption isotherms at 77 K using the Brunauer–Emmett–Teller (BET, Quick 200, Altamira Instruments) method. Hydrogen absorption behavior was investigated using a high-pressure volumetric analyzer (HPVA II, Micrometrics), under conditions appropriate to each material system.

### 2.5 Molecular dynamics (MD)

Molecular dynamics simulations were performed using LAMMPS to study hydrogen diffusion through activated carbon layers on TiFe alloy surfaces. The TiFe alloy was modeled as a polycrystalline BCC slab, while the activated carbon was represented by five porous graphene layers (SI Fig. S9). Detailed simulation parameters and structural information are provided in the SI.

## 3. Results and discussion

This section reveals the development and investigation of a composite system aimed at combining physisorption and chemisorption mechanisms for solid-state hydrogen storage. The most suitable carbon additive is first selected based on key parameters indicated in Section 3.1. Subsequently, Section 3.2 presents the impact of the chosen carbon additive on TiFe is analyzed, focusing on its effectiveness in minimizing oxides formation and enhancing hydrogen storage performance.

### 3.1 Rational selection of biomass-derived activated carbon additive (C*x*)

The CHN analysis offers insights into elemental composition of the samples, which is crucial for understanding their adsorption properties. Activated carbons typically possess a higher density of oxygen-containing functional groups relative to the char, contributing to an enhanced hydrogen storage capacity.^1^ The values given in SI Table S1 are the average of four measurements. Sample Cc displayed the highest carbon content (93.5 wt%) and the lowest oxygen content (6.3 wt%), resulting O/C atomic ratio (0.050). Sample Cg exhibited a slightly lower carbon content (90.2 wt%) and the highest oxygen content (9.1 wt%), corresponding to the highest O/C ratio (0.076) among the three. Sample Co presented intermediate values, with 91.3 wt% carbon and 8.4 wt% oxygen, yielding an O/C ratio of 0.069. While Co heteroatom content lies between that of Cg and Cc, it maintained the same H/C ratio as Cc (0.026), suggesting comparable degrees of aromaticity and structural order. The content and effect of nitrogen on hydrogen sorption can be neglected due to its low concentration, ranging from 0 to 0.1 wt%.

**Fig. 2:**
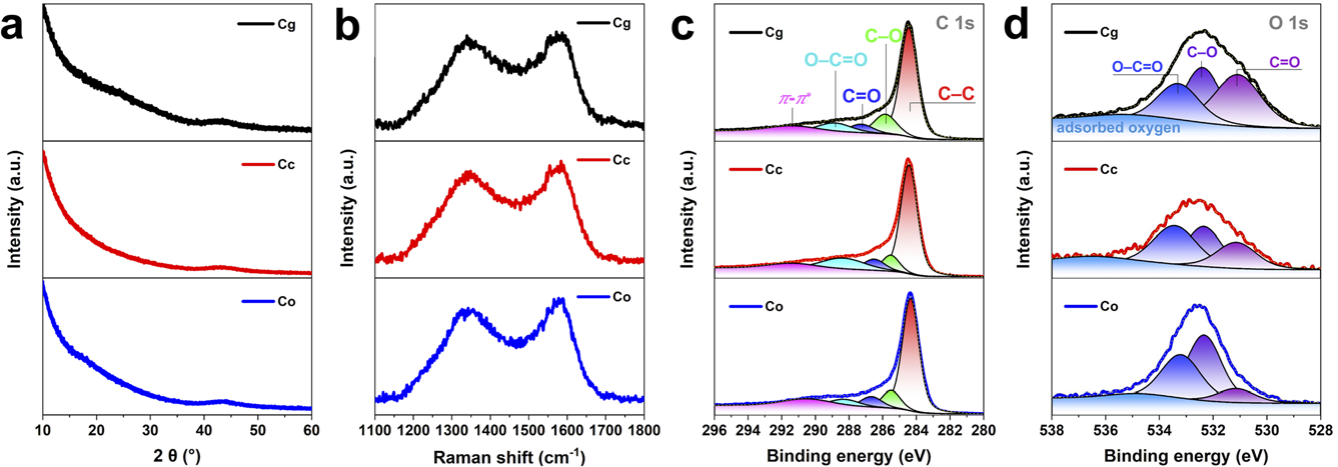
Characterization of C*x*. (a) XRD patterns. (b) Raman spectra. (c) High-resolution C 1s XPS spectra with peak fitting. (d) High-resolution O 1s XPS spectra with peak fitting.

To further probe the nature of the carbon materials XPS was performed. XPS detects the presence of elements on the surface and their bonding with each other. Wide scans were used to quantify the surface elements detected, and only carbon, oxygen, and silica, were present in detectable quantities as shown in SI Table S2. The surface carbon contents estimated from survey are 84.6 at% (Cg), 93.4 at% (Cc) and 85.3 at% (Co). With the high-resolution spectra it was possible to estimate the amount of carbon bonded to oxygen. The C 1s spectra showed a broad signal deconvoluted into five definite peaks (Fig. 2c and SI Table S3); these correspond to binding energies of 284.3–284.4 eV, characteristic of unfunctionalized carbon (C–C), C–O at 285.5–285.8 eV, C=O at 286.5–287.3 eV, O–C=O at 288.3–288.9 eV, and π–π* at 290.5–291.4 eV.^31–33^ The surface oxygen contents estimated from XPS (SI Table S2) are 12.9 at% (Cg), 6.7 at% (Cc) and 12 at% (Co). The O/C atomic ratio obtained from bulk CHNS analysis and the O (at%)/C (at%) ratio from surface sensitive XPS were compared to assess oxygen enrichment at the surface relative to the bulk. By comparing the O/C atomic ratio from bulk CHNS analysis with the O/C ratio derived from surface sensitive XPS, we assess the relative enrichment of oxygen at the material surface compared to its bulk composition. For Cg, the O/C ratio is 0.076 from CHNS and 0.153 from XPS; for Cc, 0.05 from CHNS and 0.071 from XPS; and for Co, 0.069 from CHNS and 0.141 from XPS. The close agreement between surface and bulk O/C ratios in Cc suggests a relatively uniform oxygen distribution throughout the material, whereas the elevated surface ratios in Cg and Co indicate surface enrichment of oxygen-containing species. The O 1s spectra (Fig. 2d) revealed four distinct peaks, corresponding to characteristic binding energies of oxygen-containing functional groups: a peak at 531.07–531.1 eV attributable to C=O, a peak at 532.3–532.4 eV assigned to C–O, and a peak at 533.2–533.4 eV indicative of O–C=O, and a peak at 535 eV representing the adsorbed oxygen^33,34^ (SI Table S3). Previous studies have shown that oxygen-containing functional groups, such as epoxy, hydroxyl, carbonyl, and carboxyl, partially convert graphene from sp^2^ to sp^3^ hybridization, reducing strength and conductivity, yet enhancing charge-storage performance.^35^ Furthermore, another research work^36^ indicated that oxygen-functionalized graphene models exhibit stronger hydrogen adsorption than pristine graphene, with adsorption energies (Δ*E*_abs_) following the trend –2OH > –2O > –COOH > pristine, indicating that these functional groups facilitate H_2_ stabilization.

Elemental composition analysis using EDS (SI Fig. S2) revealed the presence of carbon, oxygen, nitrogen, and minor amounts of silicon. An exception was observed in sample Cc, where silicon was absent. The presence of nitrogen is corroborated by CHN analysis, while the presence of silicon is supported by XPS data.

The XRD pattern of the biomass-derived activated carbon additives (Fig. 2a) shows a very broad peak centered at 2*θ* ≈ 22°, which is relatively featureless, indicating that the carbon has mainly amorphous nature without any graphitic domains. In addition, Dahn *et al.* used the empirical parameter (*R*), which is defined as the ratio of height of the (002) Bragg peak to the background.^37^ A larger *R* value indicates a higher degree of graphitization, which suggests a larger concentration of the parallel single layers in the carbon materials. The *R* values of Cg, Cc, and Co are close to 1, indicating very small variations in the *R* value, as also reported by Huanlei Wang *et al.* for AC-K5. This is attributed to the nearly random distribution of graphene sheets within the structure. The Raman spectra in Fig. 2b also prove the non-graphitic nature of Cg, Cc, and Co, as indicated by bands at 1342–1347 cm^−1^ and 1577 cm^−1^ from the D-peak (disordered carbon) and the G-peak (graphitic domains), respectively. The ratio of peak intensity (in terms of area) of the D-peak to G-peak (*I*_D_/*I*_G_ ratio) is 0.95 for Cg, 0.93 for Cc and 0.89 for Co. The *I*_D_/*I*_G_ ratio of 0.89–0.95 confirms the non-graphitic nature of the carbons. The increase in the *I*_D_/*I*_G_ ratio is due to the disruption of graphitic domains caused by activation at high temperatures.

**Fig. 3:**
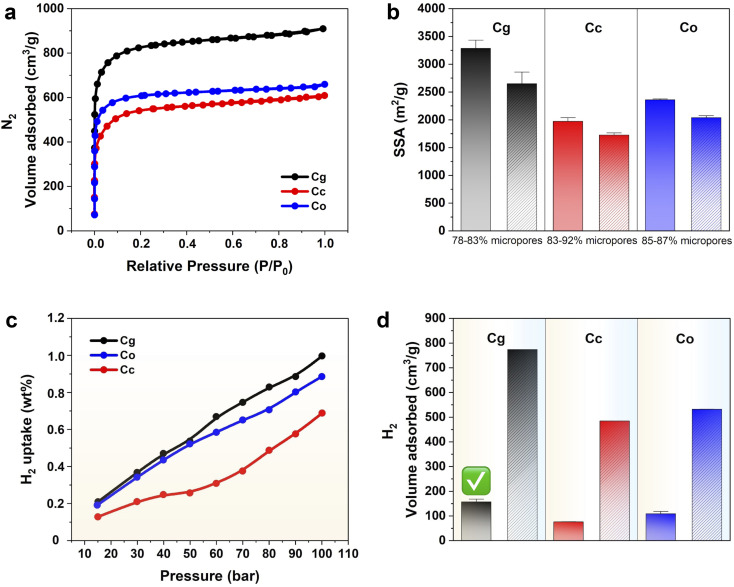
Characterization of C*x*. (a) N_2_ adsorption–desorption isotherms. (b) Bar chart showing average specific surface area and micropore surface area (dashed segments). (c) H_2_ uptake. (d) H_2_ adsorption volumes at 100 bar measured at 298 K (yellow-shaded) and 77 K (blue-shaded).

The data discussed above confirm that the carbons are compositionally pure, exhibit low levels of graphitization, and contain oxygen functionalities. The porosity of the biomass-derived activated carbons was further probed using nitrogen sorption analysis, as porosity is necessary for high hydrogen capacity. The N_2_ isotherms are presented in Fig. 3a. The behavior corresponds to an IUPAC's Type I isotherm, characteristic of microporous structured materials where adsorption occurs primarily through pore-filling mechanism. At low relative pressures, a steep rise in nitrogen uptake is observed, indicating rapid filling of micropores.^38^ This is followed by a plateau at higher pressures, where further adsorption is minimal, suggesting saturation of the micropores. The amount of nitrogen adsorbed, which reflects the material's porosity, is highest for Cg, followed by Co and then Cc. The textural properties of the carbons are summarized in SI Table S4. The average specific surface areas (Fig. 3b) from repeated syntheses are 3286 m^2^ g^−1^ for Cg, 1974 m^2^ g^−1^ for Cc, and 2362 m^2^ g^−1^ for Co, with corresponding pore volumes of 1.46 cm^3^ g^−1^, 0.87 cm^3^ g^−1^, and 1 cm^3^ g^−1^, respectively. Apart from their very high surface area and pore volume, the porous carbons feature a remarkably high level of microporosity. In sample Cg, 78–83% of the surface area is attributable to micropores, whereas in samples Cc and Co, this proportion exceeds 83%. Micropores also account for 89% of the pore volume in Cg and Cc, and contribute 0.92% in Co. A key point to note herein is that a linear relationship has been observed between the hydrogen uptake capacity and micropore volume.^37^ The pore size distribution for all three samples is similar; the carbons have mainly micropores consisting of ultra-micro-pores of size 0.63–0.65 nm (SI Table S4). Comparing our results with those from previous studies, several examples merit highlighting. Spent coffee grounds and onion peel precursors have already been used in solid-state hydrogen storage, whereas garlic peel remains unexplored in this context, making this report uniquely significant. Teng *et al.* utilized garlic-peel-derived carbon for high-performance supercapacitors, achieving specific surface areas of 2548–3887 m^2^ g^−1^, with differences in treatment and an activating agent ratio of 4 : 1.^39^ Akasaka *et al.* showed that spent coffee showed 2070 m^2^ g^−1^ prepared by different treatment.^40^ Musyoka *et al.* achieved specific surface areas ranging from 2241 to 3150 m^2^ g^−1^ from onion-peel-derived carbon through varied treatment approaches.^41^

**Fig. 4:**
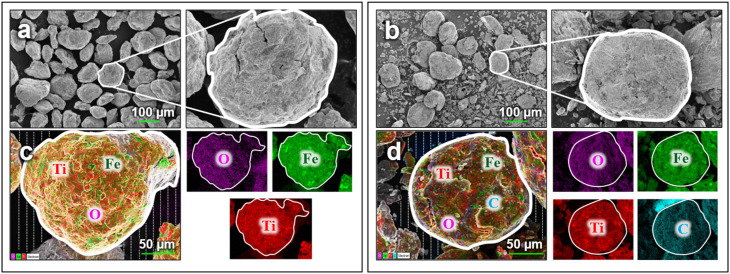
Characterization of TiFe-based materials. (a) and (c) SEM and EDS of TiFe. (b) and (d) SEM and EDS of TiFe@Cg. The dashed lines in EDS indicate the carbon tape.

Hydrogen uptake, calculated from *V*_H_2__ using SI eqn (S1), for biomass-derived activated carbons between 10 and 100 bar is shown in Fig. 3c. This pressure range was chosen to align with alloy and composite studies. Hydrogen uptake measurements at ∼0.78 bar and room temperature yielded values of 0.012 wt% for Cg, 0.024 wt% for Cc, and 0.019 wt% for Co. At 6.5 bar, the corresponding uptake values increased to 0.098 wt% (Cg), 0.074 wt% (Cc), and 0.098 wt% (Co). Notably, Cg and Co exhibited comparable uptake capacities between 6.5 and 50 bar (Fig. 4c), diverging at higher pressures. Overall, significant hydrogen uptake at room temperature is observed only at moderate pressures, suggesting that alloy performance may be enhanced under these conditions in subsequent applications or analyses. Additionally, the *V*_H_2__ at 100 bar was compared across various carbon precursors to evaluate their maximum hydrogen storage capacities under extreme conditions, high pressure, and cryogenic temperature (blue-shaded bar) (Fig. 3d). This comparison enabled the selection of the most promising candidate for further testing at room temperature (yellow-shaded bar). Among the precursors tested, Cg exhibited the highest uptake and was therefore selected for further modification with TiFe. At 77 K, Cg adsorbs 6.96 wt% hydrogen at 100 bar (SI Fig. S7), representing, to our knowledge, among the highest reported values.

The surface morphology and structural properties of the biomass-derived activated carbon was analyzed using SEM images (SI Fig. S3). These images reveal distinct morphological transformations induced by the activation process. Despite being prepared using the same method, the samples exhibit distinct structural features, highlighting the strong influence of precursor type on final morphology. Garlic peel-derived activated carbon (SI Fig. S3a) shows a uniform and well-developed porous structure with a rough, continuous surface, suggesting efficient structural transformation. In contrast, coffee ground-derived carbon (SI Fig. S3b) presents a more irregular and fragmented morphology with visible cavities and a sponge-like appearance. Onion peel-derived carbon (SI Fig. S3c) reveals a compact, wrinkled surface with less pronounced porosity and a more uneven texture. The obtained porosity is mainly a result of the reactions between potassium hydroxide and the carbonaceous material during pyrolysis.^38^ KOH initially reacts to produce potassium carbonate, which subsequently decomposes into metallic potassium and CO_2_ at high temperatures. The formation of gaseous byproducts generates voids and channels within the carbon matrix, thereby resulting in a highly porous structure. The increased porosity greatly improves the pore volume and surface area of the activated carbon, providing numerous active sites for the adsorption of hydrogen molecules. Furthermore, the interconnected pore network facilitates diffusion paths for gases, hence ensuring high adsorption efficiency.^38^

An additional important consideration is the powder density of the samples. Cg exhibited the lowest density at 2.0412 ± 0.0057 g cm^−3^, compared to 2.3417 ± 0.0030 g cm^−3^ for Cc and 2.1330 ± 0.0022 g cm^−3^ for Co. This indicates that Cg is significantly lighter than the other precursors, consistent with the direct relationship between density and mass.

### 3.2 TiFe-based materials characterization

Following the synthesis method reported in literature for TiFe,^30^ we reproduced the conventional TiFe system and subsequently modified the process to introduce a rationally selected biomass-derived carbon additive. To evaluate the characteristics of TiFe and the effect of the rationally selected biomass-derived activated carbon on the alloy, further characterization analyses were performed.

ICP analysis was further performed to verify the stoichiometric ratio of Ti and Fe in the synthesized alloy. The results confirmed a composition approaching the targeted 1 : 1 atomic ratio, with measured concentrations of 111.8 ppm for Ti and 126.7 ppm for Fe. Further characterizations of the materials were performed with SEM (Fig. 4a and b) and EDS (Fig. 4c and d), revealing both the surface morphology and elemental distribution of TiFe and TiFe@Cg. As reported in previous studies,^27,42^ extended ball milling can lead to the agglomeration of fine particles. This phenomenon is evident in Fig. 4a, where larger particles appear to be formed from smaller constituents, as suggested by the presence of visible intergranular cracks. In contrast, such features are less prominent in the TiFe@Cg composite, likely due to the dispersion of carbon across the alloy surface. This carbon coating led to a reduction in the oxygen signal in the EDS spectra (SI Fig. S4), indicating diminished surface oxidation. For pristine TiFe, titanium and iron were present at 37.3 wt% and 37.1 wt%, respectively, with the remaining fraction attributed primarily to surface oxygen and the underlying carbon tape used during sample preparation. In the TiFe@Cg composite, the concentrations of Ti and Fe decreased to 26.5 wt% and 25.6 wt%, respectively, while the carbon content increased to 29.8 wt%, a value that includes both the added carbon coating and the carbon tape contribution. These results are consistent with the successful integration of carbon into the alloy and further support the role of carbon in shielding the alloy from oxidation. Consistent with previous reports, the carbon phase is known to enhance hydrogen storage capacity, kinetics, while simultaneously acting as a protective barrier, mitigating further oxidation of the alloy surface.^27,28,43^

**Fig. 5:**
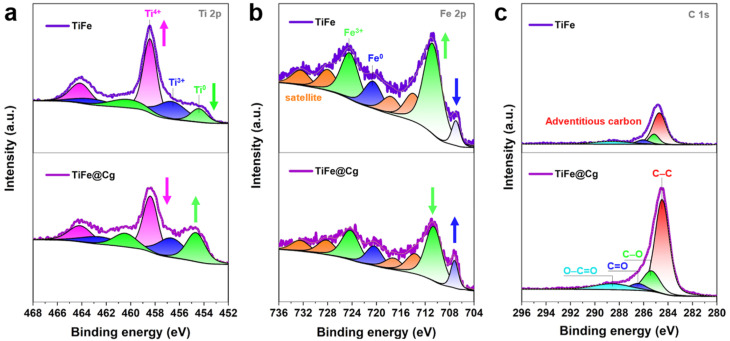
Characterization of TiFe-based materials. (a) High-resolution Ti 2p XPS spectra with peak fitting. (b) High-resolution Fe 2p XPS spectra with peak fitting. (c) High-resolution C 1s XPS spectra with peak fitting.

To demonstrate that carbon unambiguously acts as a barrier against oxidation, and to gain insight into the possible chemical interactions between TiFe and Cg, XRD and XPS analyses were performed. The XRD pattern of the TiFe@Cg composite is virtually identical to that of pristine TiFe (SI Fig. S5), indicating that the introduction of carbon does not disrupt the crystal structure of the intermetallic phase. This preserved diffraction profile implies a lack of significant chemical interaction or structural integration between TiFe and the carbon matrix. As shown in Fig. 5, the overall Ti 2p and Fe 2p signal intensities of TiFe@Cg are lower than those of pristine TiFe, indicating that the carbon coating may partially cover the surface of the TiFe alloy and attenuate the signals. Notably, the atomic percentages of metallic titanium and iron are higher in TiFe@Cg compared to TiFe, while the corresponding oxide forms exhibit lower atomic percentages (SI Table S3). Specifically, in TiFe, Ti^0^ is observed at 454.4 eV and 460.2 eV (ref. 44) with atomic percentages of 8.31 at% and 11.05 at%, respectively. In contrast, TiFe@Cg shows Ti^0^ peaks at 454.6 eV and 460.4 eV with higher atomic percentages of 19.12 at% and 10.94 at%, respectively. Additionally, TiFe exhibits Ti^4+^ peaks at 458.4 eV and 464.1 eV (ref. 44 and 45) which were assigned to Ti 2p_1/2_ and 2p_3/2_ electrons in Ti^4+^ corresponding to TiO_2_,^44,46,47^ with atomic percentages of 37.78 at% and 15.47 at%, respectively. Conversely, TiFe@Cg displays Ti^4+^ peaks at 458.3 eV and 464.1 eV, corresponding to lower atomic percentages of 29.24 at% and 12.97 at%. Additionally, for both TiFe and TiFe@Cg, two distinct peaks centered at 456.6 eV and 463.1 eV (456.6 eV and 462.5 eV for TiFe@Cg) are observed, which can be attributed to Ti^3+^ species, indicative of the formation of a surface Ti_2_O_3_ phase.^28,48,49^

A similar trend is observed in the Fe 2p XPS spectra; In TiFe and TiFe@Cg, binding energy peaks were observed at 710.8 and 710.6 eV, and at 724.4 and 724.2 eV, respectively, indicative of the Fe^3+^ oxidation state corresponding to the Fe_2_O_3_ phase.^28,44^ Two additional binding energy peaks at 720.5 and 720.3 eV, as well as at 706.9 and 707.2 eV, were observed for TiFe and TiFe@Cg, respectively, indicating the presence of metallic iron.^44^ For detailed binding energy values and atomic compositions, refer to SI Table S3. These results indicate that the oxides are only partially removed, yet this still enhances hydrogen uptake. Its persistence is likely due to the carbon's microporous structure: although it serves as a barrier, pores of ∼0.65 nm remain large enough to permit oxygen penetration. As shown in the C 1s spectra (Fig. 5c), no evidence of chemical bonding between TiFe and carbon is observed, suggesting that the carbon is merely coated on the TiFe surface and is likely held in place by cracks and defects generated during the ball-milling process.

### 3.3 Hydrogen storage TiFe alloy and composite

**Fig. 6:**
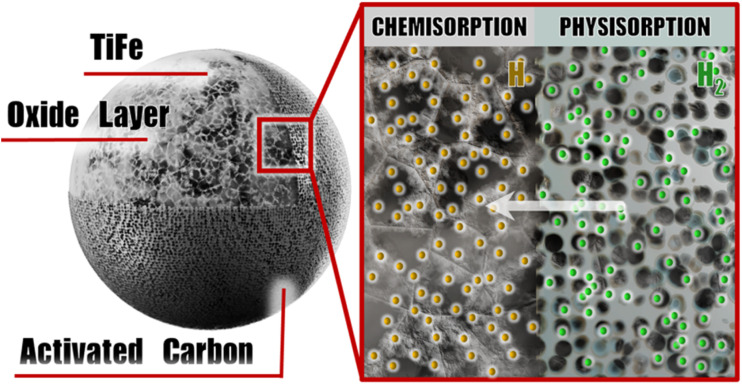
Schematic illustration of the proposed hydrogen sorption mechanism (for illustrative purposes, Blender software).

High hydrogen storage capacity and fast reaction kinetics are key requirements for practical hydrogen storage materials. In the TiFe@Cg composite, hydrogen molecules initially encounter the porous activated carbon layer, where they may be physisorbed or diffuse through the micropores to reach the underlying TiFe surface, as shown in Fig. 6.

During physisorption, molecular hydrogen interacts weakly with the carbon surface *via* van der Waals forces, with no significant activation energy barrier.^50,51^ Thus, achieving a significant hydrogen storage necessitates cryogenic conditions due to the low adsorption enthalpy (typically <10 kJ mol^−1^),^50,52^ limiting its application at room temperature. However, Cheng *et al.* presented comparative results for various materials at room temperature, showing that around 1 wt% H_2_ can be achieved at moderate pressures ranging from 15 to 270 bar.^20^ Therefore, the moderate pressure range examined in this study (10–100 bar) facilitates significant hydrogen uptake by porous materials and alloy saturation, enabling clear observation of carbon's effect on the alloy.

Upon diffusing through the carbon matrix, hydrogen molecules reach the TiFe alloy surface, where they undergo dissociative chemisorption. This process involves the cleavage of the H–H bond and insertion of hydrogen atoms into interstitial sites within the TiFe lattice, forming a metal hydride.^53^ The detailed mechanism, as described by Sujan *et al.*, involves the initial dissolution of hydrogen into the TiFe lattice, forming a solid solution known as the α-phase, which is characterized by a steep increase in hydrogen concentration. Upon reaching the solubility limit of the α-phase, a first-order phase transformation occurs, resulting in the precipitation of TiFe hydride in the β-phase at a nearly constant plateau pressure (SI Fig. S6). Continued hydrogen absorption causes a sharp pressure increase within the single β-phase region until the formation of the γ-phase at higher hydrogen concentrations and pressures, producing a second plateau at elevated pressure. The γ-phase corresponds to the fully hydrogenated state of TiFe. The research focuses on the β-phase to γ-phase transition, examining TiFe under full hydrogen loading to evaluate its maximum hydrogen uptake and the effects of rationally selected additives on its performance. This study focuses on moderate pressures below 100 bar, within which the β-phase is stable from 10 to 90 bar, and the γ-phase emerges between 90 and 100 bar.

**Fig. 7:**
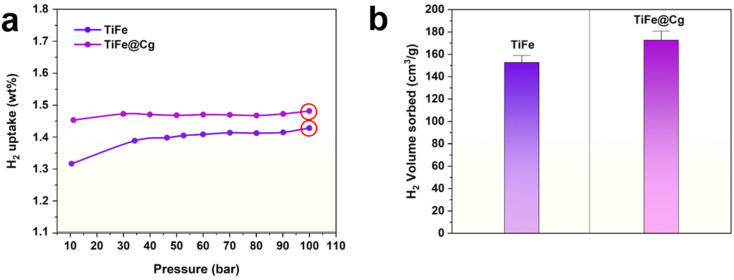
Hydrogen uptake performance of TiFe-based materials at 298 K. (a) Enlarged view highlighting the β and γ phase regions of hydrogen uptake. (b) Bar chart displaying the reproducible maximum hydrogen uptake at 100 bar.

The previously reported values for TiFe-based systems have reported that absorption plateau pressures initiate at ranges of 15–20 bar, 4–9 bar, and 10–20 bar for hydrogen storage capacities of 1.1 wt% H_2_,^45^ 1.3 wt% H_2_,^54^ and up to 1.5 wt% H_2_,^9^ respectively. As shown in Fig. 7a, TiFe produced by ball milling under inert atmosphere exhibits a hydrogen uptake of 1.31 wt% H_2_ at 10 bar and 298 K. In contrast, TiFe modified with a carbon additive exhibits a 10.7% increase in uptake under identical conditions, achieving 1.45 wt% H_2_. At moderate pressures up to 100 bar, TiFe and TiFe@Cg displaying hydrogen capacities of 1.43 wt% H_2_ and 1.48 wt% H_2_, respectively. The enhancement in hydrogen uptake for the carbon-modified sample is partially attributed to the hydrogen adsorption capability of the porous carbon itself:^28^ 110 mg of the selected carbon material (Cg) can independently store 0.99 wt% H_2_ at 100 bar and 298 K (Fig. 3c). Given that only 1 wt% carbon was incorporated into the TiFe@Cg composite, the remaining enhancement is likely due to other synergistic effects, such as suppression of surface oxide formation. Moreover, the hydrogenation kinetics appear modestly improved for the TiFe@Cg composite (SI Fig. S8), suggesting a secondary benefit of the carbon additive, potentially through enhanced surface reactivity or improved diffusion pathways. A plateau was reached after 140 min for TiFe, and after 139 min for TiFe@Cg. Importantly, both TiFe and TiFe@Cg maintain similar hydrogenation curve profiles within 10–100 bar, consistent with their identical synthesis method and the inert role of carbon (*i.e.*, no chemical bonding to the alloy). This similarity underscores the reproducibility and uniformity of the preparation method. Additionally, identical samples were measured independently at two separate institutions using the same hydrogen sorption instrumentation. As illustrated in Fig. 7b, the results demonstrate excellent agreement, while reaffirming the enhancement in hydrogen capacity at moderate pressures due to carbon addition.

### 3.4 Molecular dynamics (MD)

MD simulations were performed to analyze the molecular interactions during physisorption of H_2_ on the TiFe@Cg material. X-ray diffraction of TiFe covered by activated carbon showed that carbon atoms do not disturb the crystal structure of the intermetallic phase, indicating that there is no significant chemical interaction between TiFe and the carbon matrix. Also, it is known from the study by Mahmoud *et al.* that during the process of physical adsorption, molecular hydrogen weakly interacts with the carbon surface through van der Waals forces, without creating a significant activation energy barrier. Therefore, in this MD study, the interaction of hydrogen atoms with carbon atoms,^55,56^ as well as iron and titanium atoms in the alloy,^57^ is determined by the LJ 12–6 potentials. The LJ potential parameters used in this study are taken from the study by Mishra *et al.*, and the potentials between unlike atoms were calculated using the Lorentz–Berthelot mixing rule.^57^ The cutoff distance was set to 12 Å to include short-range electrostatic interactions and van der Waals forces.^58^ For titanium and iron atoms, the interactions within the alloy are described by the modified embedded atom method (MEAM).^59^

To ensure structural stability and relaxation of the system, energy minimization was applied to the simulation cell using the conjugate gradient method with strict energy and strength tolerances set at 10^−10^ eV and 10^−12^ eV Å^−1^. The Nose–Hoover thermostat was used for maintaining the constant temperature condition. Newton's equations of motion were integrated using the velocity Verlet algorithm with a time step of 1 fs.^56^ Periodic boundary conditions were applied in *x* and *y* directions, while reflective walls were applied in the *z*-direction with vacuum slabs (thickness of 20 Å each) at the top and the bottom of the box. In the production run, an NVT canonical ensemble at 298 K with a time step of 1 femtosecond (0.001 in metallic units) was used for 5 ns for both the TiFe alloy–hydrogen system and the TiFe alloy–activated carbon–hydrogen system,^58^ see SI Fig. S10 in the initial and final state.

**Fig. 8:**
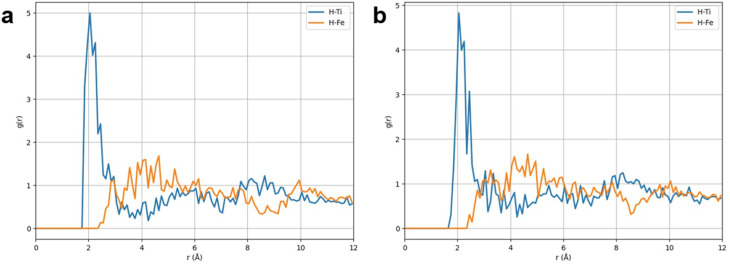
Radial distribution function (RDF) of hydrogen atoms relative to Ti and Fe atoms in the alloy slab. (a) TiFe and (b) TiFe–activated carbon.

As a result of the MD simulations, radial distribution function (RDF) calculations were carried out to evaluate the local coordination of hydrogen atoms with Ti and Fe in the alloy slab for both TiFe and TiFe–activated carbon systems (Fig. 8). RDF of hydrogen atoms relative to Ti and Fe atoms was computed from the equilibrated snapshot (see SI part) with wrapped coordinates for each system.

For the TiFe system, the RDF shows a pronounced first peak at ∼2.0 Å, characteristic of strong direct coordination of hydrogen with alloy sites, particularly with Ti atoms, which contribute more strongly than Fe to hydrogen binding. Beyond the first coordination shell, both H–Ti and H–Fe correlations decay smoothly toward unity after ∼5 Å, indicating a transition to a uniform distribution as the local structural order diminishes. The sharpness and height of the first peak emphasize the strength and well-defined nature of hydrogen–metal interactions in the unmodified alloy. In the TiFe–activated carbon system, the first peak is weaker in intensity and slightly shifted to longer distances compared to pure TiFe, reflecting a reduction in direct hydrogen coordination with Ti and Fe sites under the influence of the carbon phase. A distinct additional peak emerges at ∼2.65 Å, exceeding the corresponding values in the unmodified alloy. This feature highlights hydrogen redistribution into the carbon matrix, where adsorption sites provide an alternative binding environment. Thus, while hydrogen–metal interactions are weakened in the carbon-modified system, the presence of carbon introduces new adsorption pathways that significantly reshape the local hydrogen environment.

Moreover, a quantitative analysis was performed to calculate the amount of hydrogen molecules absorbed in TiFe alloy crystal structure by the Center of Mass (COM) calculated over a 5 ns trajectory using unwrapped coordinates atomic positions (*x*_u_, *y*_u_, *z*_u_). The COM calculations were restricted exclusively to the TiFe alloy region, ensuring that the reported dynamics represent intrinsic hydrogen motion within the metallic matrix rather than contributions from the porous carbon phase. As a result of COM calculation of the hydrogen molecules diffused in the region of the TiFe alloy slab was mean square displacement (MSD) analysis.^57,60,61^ The MSD trend shows faster diffusion in the TiFe alloy, while the TiFe–activated carbon system exhibits reduced mobility due to hydrogen being partially retained by the carbon matrix and therefore initial delay of hydrogen molecules reaching the alloy slab, but same increasing trend, see in SI Fig. S11.

## 4. Conclusion

In summary, we demonstrate a sustainable and effective strategy to improve the hydrogen storage performance of TiFe alloys through the use of a biomass-derived activated carbon additive produced from agricultural waste. Among the tested precursors, garlic peel-derived activated carbon exhibited optimal characteristics, facilitating enhanced hydrogen sorption capacity and kinetics with minimal additive loading (1 wt%). Experimental findings indicate an increase in hydrogen uptake from ∼1.4 to 1.5 wt% H_2_ and accelerated kinetics under ambient conditions, while atomistic simulations clarify the influence of the carbon phase on hydrogen diffusion characteristics. The molecular dynamics simulations reveal that the addition of activated carbon to TiFe alloys modifies the local hydrogen environment by weakening direct hydrogen–metal interactions and introducing new adsorption sites, as evidenced by RDF analysis. The MSD results further show that while hydrogen diffusion within the TiFe alloy remains largely unchanged in the long term, the activated carbon layer induces a delayed entry of hydrogen due to initial retention within the carbon matrix. Together, these findings highlight the dual role of carbon in altering both the spatial distribution and initial transport dynamics of hydrogen in TiFe-based systems.

This work not only advances the design of improved hydrogen storage materials, but also offers a scalable and environmentally responsible method through the valorization of agricultural waste. The combined experimental and computational insights provide a foundation for the rational design of hybrid metal–carbon systems with tailored interfacial properties. Further research into such systems is necessary to deepen our understanding of hydrogen–material interactions and to accelerate the development of next-generation hydrogen storage technologies.

## Author contributions

Madina Kalibek: conceptualization, methodology, investigation, data curation, writing – original draft. Nurbolat Issatayev: conceptualization, methodology. Aigerim Ospanova: investigation, writing – review & editing. Talgat Orazbek: investigation. Ayaulym Amankeldiyeva: software, data curation. Mirat Karibayev: software, data curation. Vladislav Kudryashov: supervision, methodology. Aitkazy Kaisha: supervision. Dhawal Shah: supervision, writing – review & editing. Nurxat Nuraje: supervision, writing – review & editing, funding acquisition.

## Conflicts of interest

The authors declare that they have no known competing financial interests or personal relationships that could have appeared to influence the work reported in this paper.

## Supplementary Material

RA-016-D5RA07863F-s001

## Data Availability

The data supporting this article have been included as part of the supplementary information (SI). Supplementary information is available. See DOI: https://doi.org/10.1039/d5ra07863f.
