# Supplementary material for: Enhancing hydrogen uptake in TiFe at moderate pressures via rationally designed biomass-derived carbon additives
Source: RSC Adv. 2026 Jan 2;16(1):687–99. doi: 10.1039/d5ra07863f (PMC12757930; doi:10.1039/d5ra07863f)
Supplement: RA-016-D5RA07863F-s001 [file RA-016-D5RA07863F-s001.pdf]

Supplementary Information (SI) for RSC Advances.
This journal is © The Royal Society of Chemistry 2025

Rationally Selected Biomass-Derived Carbon Additive Enhances Hydrogen Uptake in TiFe at Moderate Pressures

Authors: Madina Kalibek[1,2], Nurbolat Issatayev[2], Aigerim Ospanova[1,2], Talgat Orazbek[1], Ayaulym Amankeldiyeva[1], Mirat Karibayev[1], Vladislav Kudryashov[1], Aitkazy Kaisha[1], Dhawal Shah[2], Nurxat Nuraje[1,2]*

* N. Nuraje, nurxat.nuraje@nu.edu.kz | [1] Renewable Energy Lab, National Laboratory Astana, Nazarbayev University, Astana 010000, Kazakhstan. [2] Department of Chemical and Materials Engineering, School of Engineering and Digital Science, Nazarbayev University, Kabanbay Batyr Ave. 53, Astana 010000, Kazakhstan.

Supplementary Part

Cx: biomass-derived activated carbon additive
TiFe@Cg: Composite

Hydrogen Uptake Measurements

Hydrogen uptake capacity of the materials was evaluated using a HPVA II volumetric adsorption analyzer with high-purity hydrogen gas (99.999%). Prior to measurement, all samples underwent degassing under vacuum to eliminate adsorbed contaminants and moisture. Adsorption isotherms were recorded at ambient temperature (298 K) over a pressure range of 10-100 bar. Additionally, biomass-derived activated carbon materials were examined at 77 K using a liquid nitrogen bath to determine its maximum hydrogen storage capacity. The HPVA II system provided quantitative adsorption data, including the volume of hydrogen adsorbed per gram of sample (V_{H_2}, cm^3/g) and the corresponding hydrogen uptake expressed as weight percentage ($wt\%\ H_2$). While V_{H_2} was obtained directly from the instrument, the $wt\%\ H_2$values were derived according to Equation 1.

$$wt\%\ H_2 = \left(\frac{V_{H_2} \times M_{H_2}}{V_m} \right) \times 100 \tag{1}$$

where V_{H_2} = volume adsorbed by sample (cm^3/g)
M_{H_2} = molar mass of hydrogen gas = 2.0159 g/mol
V_m = molar volume of hydrogen at STP = 22.414 cm^3/mol

Supplementary Part. Tables

Table S1 | Elemental composition and atomic ratios of Cx (average of four measurements)

Sample	C (wt%)	H (wt%)	N (wt%)	O (wt%)	(O/C)[a]	(H/C)[a]
**Cg**	90.2	0.3	0.1	9.1	0.076	0.039
**Cc**	93.5	0.2	0	6.3	0.050	0.026
**Co**	91.3	0.2	0.1	8.4	0.069	0.026

[a]Atomic ratio

Table S2 | XPS survey data of Cx and TiFe and composite

Sample	Spectra	Peak Position (eV)	(at%)
**Cg / Cc / Co**	**C 1s**	284.97 / 284.9 / 284.9	84.59 / 93.35 / 85.25
	**O 1s**	532.7 / 532.66 / 532.38	12.99 / 6.65 / 12.07
	**Si 2p**	103.8 / - / 103.04	2.42 / 0 / 2.67
**TiFe / TiFe@Cg**	**Ti 2p**	458.18 / 458.04	16.98 / 8.02
	**Fe 2p**	711.2 / 711.05	10.11 / 5.72
	**O 1s**	530.24 / 530.84	39.05 / 18.8
	**C 1s**	285.07 / 284.95	26.47 / 62.13

Table S3 | High-resolution XPS peak deconvolution of the C 1s, O 1s, Ti 2p, and Fe 2p spectra of Cx and TiFe and composite

Sample	Spectra	Label	Peak Position (eV)	(at%)
**Cg / Cc / Co**	**C 1s**	C–C	284.44 / 284.43 / 284.33	59.74 / 58.6 / 61.96
		C–O	285.83 / 285.51 / 285.48	14.33 / 8.97 / 10.68
		C=O	287.27 / 286.49 / 286.66	6.68 / 7.79 / 7.67
		O–C=O	288.86 / 288.38 / 288.3	10.17 / 16.23 / 7.62
		π-π^*	291.36 / 291.37 / 290.51	9.09 / 8.41 / 12.07
	**O 1s**	C=O	531.07 / 531.13 / 531.14	37.74 / 22.02 / 10.58
		C–O	532.39/ 532.34 / 532.33	31.19 / 29.45 / 46.97
		O–C=O	533.3 / 533.43 / 533.2	25.27 / 36.46 / 36.51
		Adsorbed oxygen	535.28 / 536.47 / 534.85	5.8 / 12.06 / 5.93
**TiFe / TiFe@Cg**	**Ti 2p**	**Ti^{4+}**	458.4 / 458.37	37.78 / 29.24
			464.12 / 464.12	15.47 / 12.97
		**Ti^{3+}**	456.63 / 456.65	19.42 / 18.38
			463.18 / 462.55	7.97 / 9.35
		**Ti^{0}**	454.4 / 454.65	8.31 / 19.12
			460.21 / 460.42	11.05 / 10.94
	**Fe 2p**	**Fe^{3+}**	710.78 / 710.59	34.56 / 29.91
			724.39 / 724.24	18.58 / 17.86
		**Fe^{0}**	706.96 / 707.19	4.34 / 6.93
			720.51 / 720.32	10.54 / 10.9
	**C 1s**	**C–C**	284.71 / 284.48	61.85 / 66.61
		**C–O**	285.15 / 285.36	14.92 / 17.2
		**C=O**	286.03 / 286.45	8.35 / 5.64
		**O–C=O**	288.61 / 288.56	14.87 /10.55

Table S4 | Surface area and porosity characteristics of Cx

Sample	S_{BET} (m^2/g)	S_{micro} (m^2/g)	V_t (cm^3/g)	V_{micro} (cm^3/g)	D_{HK} (nm)
Cg	3286 ±146.872	2650±292.489	1.46±0.056	1.31±0.05	0.655±0.009
Cc	1974±64.79	1728±37.457	0.87±0.065	0.78±0.042	0.637±0.016

Co	2362±14.641	2040±32.556	1±0.015	0.92±0.01	0.645±0.055

The specific surface area (S_{BET}) was calculated using the Brunauer-Emmett-Teller (BET) method. The microporous surface area (S_{micro}) was determined by the t-plot method. V_t represents the total pore volume. The micropore volume (V_{micro}) was determined using the Horvath-Kawazoe (HK) method. D_{HK} denotes the most frequent pore diameter obtained by the HK method.

Supplementary Part. Figures

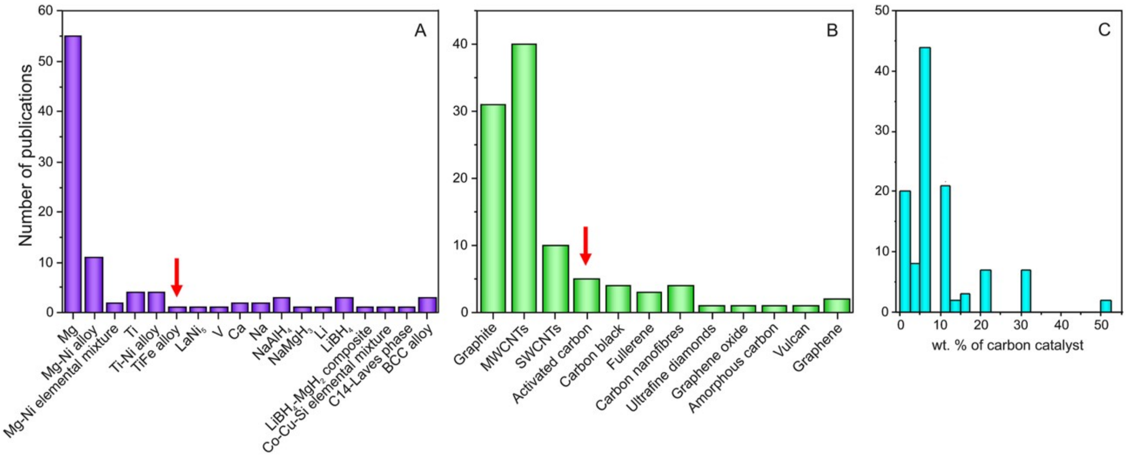

Fig. S1 | Number of publications in which: (a) a specific hydrogen storage material was catalyzed by a carbon additive; (b) a specific carbon additive was used to catalyze the hydrogen sorption/desorption reaction; (c) a specific amount of carbon additive was used. Red arrows indicate the hydrogen storage material and carbon additive used in this study. This figure is retrieved from [27].

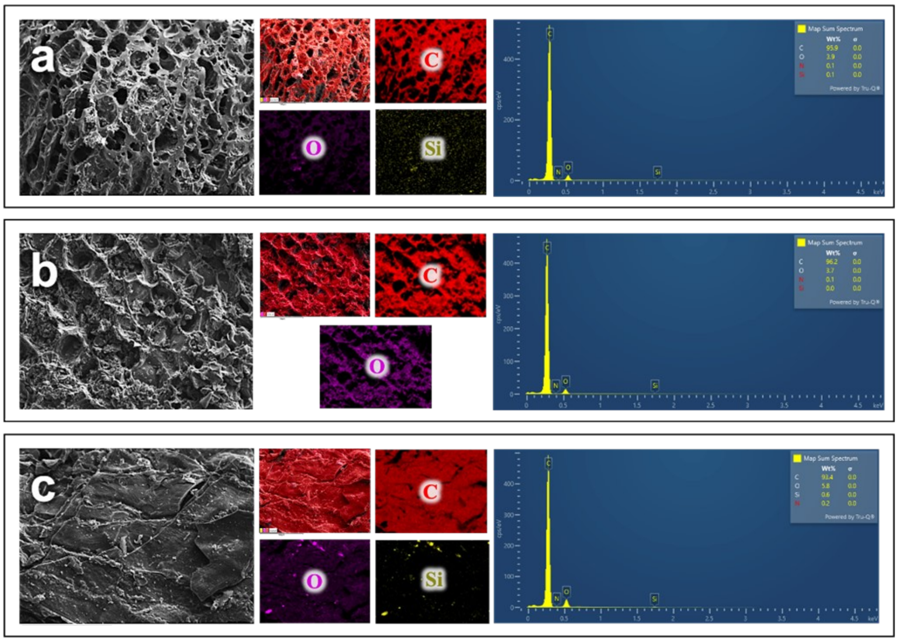

where **a**, Cg: C (95.9 wt%), O (3.9 wt%), N (0.1 wt%), Si (0.1 wt%); **b**, Cc: C (96.2 wt%), O (3.7 wt%), N (0.1 wt%), Si (0 wt%); **c**, Co: C (93.4 wt%), O (5.8 wt%), N (0.2 wt%), Si (0.6 wt%)

Fig. S2 | EDS spectra and elemental mapping of Cx.

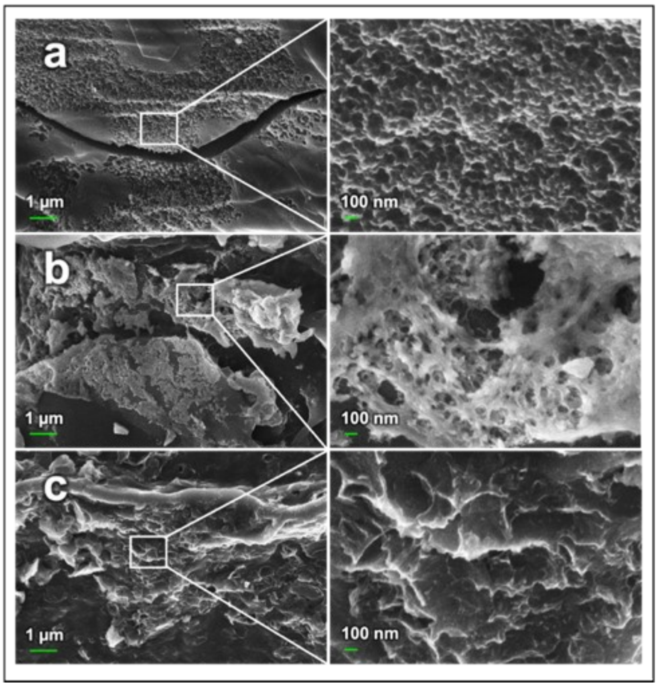

Fig. S3 | SEM images of Cx: **a** Cg (low and high magnification), **b** Cc (low and high magnification), and **c** Co (low and high magnification).

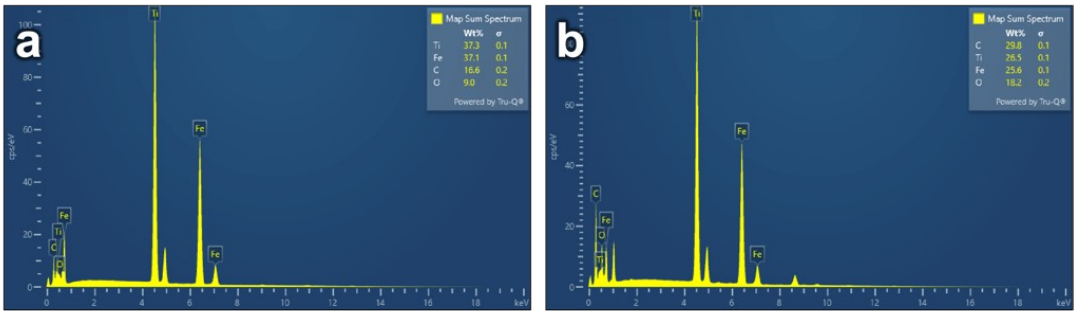

where **a**, TiFe: Ti (37.3 wt%), Fe (37.1 wt%), C (16.6 wt%), O (9 wt%); **b**, TiFe@Cg: Ti (26.5 wt%), Fe (25.6 wt%), C (29.8 wt%), O (18.2 wt%)

Fig. S4 | EDS of TiFe and composite.

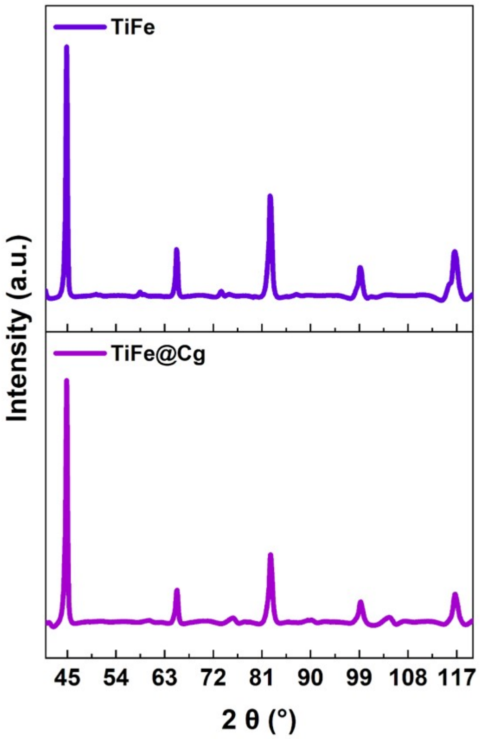

Fig. S5 | XRD patterns of TiFe-based materials after 1 cycle.

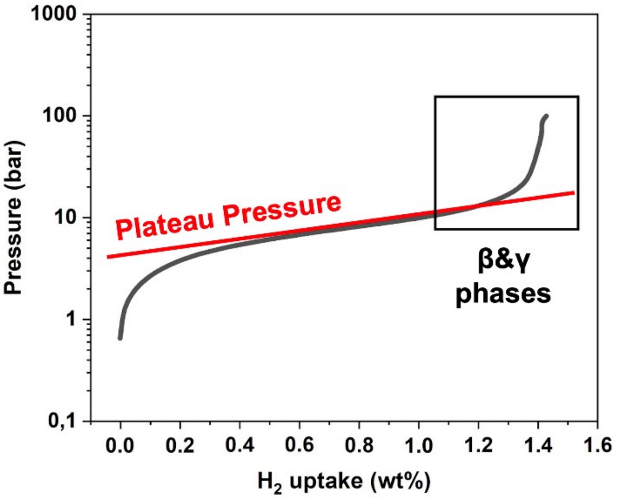

Fig. S6 | Full hydrogen absorption curve of the TiFe-based alloy.

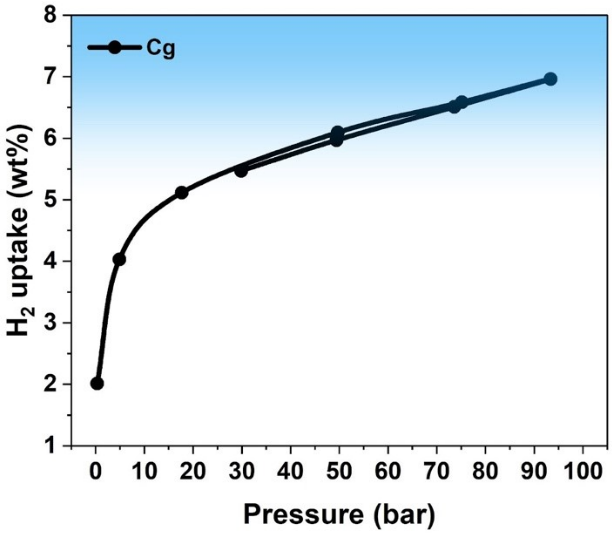

Fig. S7 | Hydrogen uptake of Cg at 77K.

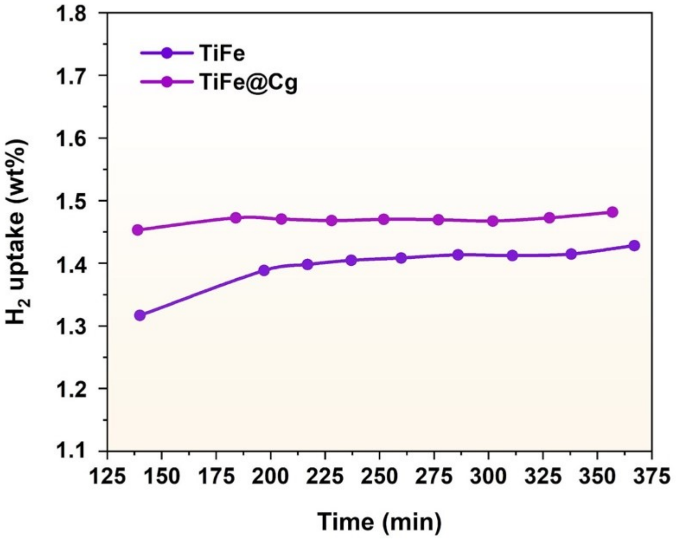

Fig. S8 | Kinetics: Hydrogen uptake versus time for TiFe-based materials.

Molecular Dynamics (MD)

A large-scale atomic-molecular massively parallel simulator (LAMMPS)[4] as used to study the diffusion of hydrogen gas through activated carbon layers on the surface of TiFe alloy to understand the mechanism of hydrogen absorption improvement when adding activated carbon using molecular dynamics (MD) simulation method. In order to construct the TiFe alloy polycrystalline system and the activated carbon, which is graphite structured material with pores (defects in structure), the open-source molecular editor and visualization software - Avogadro 1.99.0 was utilized[5].

The TiFe alloy was considered as a polycrystal with a tetraauricupride structure crystallizing in the cubic space group $Pm\bar{3}m$. The Ti is bonded to eight equivalent Fe atoms in a body-centered cubic (BCC) lattice. The length of all Ti-Fe bonds is 2.54 angstroms (Å)[6]. The polycrystalline structure of TiFe alloy, which has a thickness of 77 Å, was builded with 539 titanium and 539 iron atoms in BCC lattice represented as a solid slab. The distance between the TiFe alloy and the activated carbon was set to 5Å.

Considering the fact that the carbon content is about 90wt% from this experimental study, we made the assumption that oxygen atoms will not be present in the structure of activated carbon. Activated carbon or simply graphite with pores consists of graphene layers in which the carbon atoms are arranged in a hexagonal structure with an interatomic distance of 1.42 Å. The atoms interact through atomistic covalent bonds via sp^2-hybridized electrons, where the angle between them is 120°. The Weak van der Waals forces hold these carbon layers in an AB-type sequence along the thickness, where the interlayer distance is approximately 3.35 Å[7].

In order to create pores the carbon atoms were deleted in the structure within a diameter of 6.5 Å and with a total number of carbon atoms of 3743. The choice of 5 layers is described in the study by Jiang et al. (2015)[8], where bilayer graphene exhibits maximum hydrogen storage per added layer, since attractive van der Waals interactions act only between adjacent layers, while repulsive forces quickly weaken at large distances. Four layers have the largest number of stabilizing interactions, but adding a fifth layer ensures full convergence, since the contribution of more distant layers becomes negligible. Thus, five layers can be considered an optimal balance between maximizing storage and avoiding unnecessary structural complexity. Visualization of the activated carbon structure is presented using the Visual Molecular Dynamic (VMD) program[9] see Fig.2 (a-b).

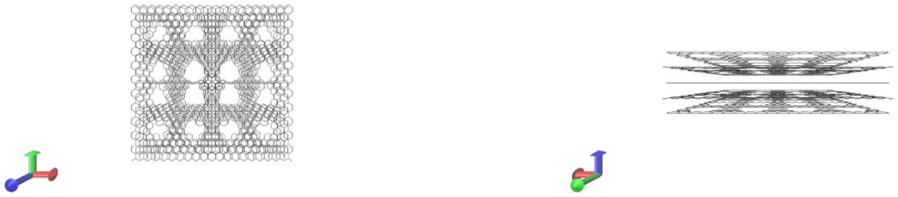

Fig. S9 | Activated carbon structure (5 graphene layers) with pore size 0.65 nm **a**, Perspective view. **b**, Projection along the z-axis.

The diffusion process is modeled using 200 hydrogen molecules in a region above activated carbon and TiFe alloy. The Pakmol software[10] was used to combine all the structures into one system for 1 case with the TiFe alloy-Hydrogen system and the 2 case with the TiFe alloy-Activated carbon- Hydrogen system in boxes with dimensions (49x49x157) Å^3 and (49x49x190) Å^3, respectively.

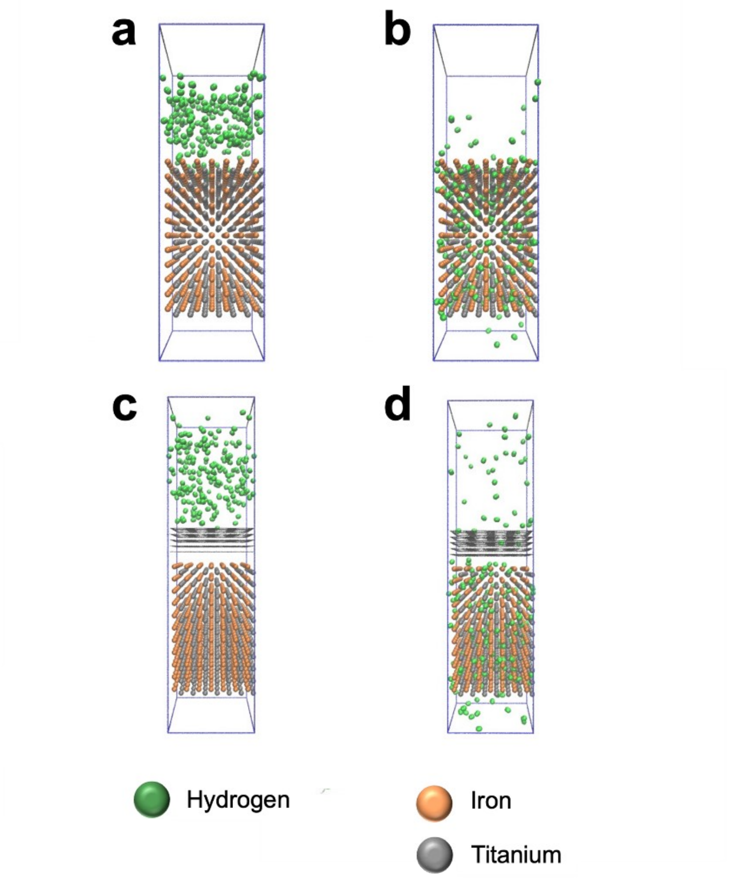

Fig. S10 | **a**, Simulation box of the TiFe–H system at 0 ns. **b**, Simulation box of the TiFe–H system at 5 ns. **c**, Simulation box of the TiFe–activated carbon–H system at 0 ns. **d**, Simulation box of the TiFe–activated carbon–H system at 5 ns. Atoms are colored as follows: hydrogen (green), carbon (black), iron (orange), and titanium (gray).

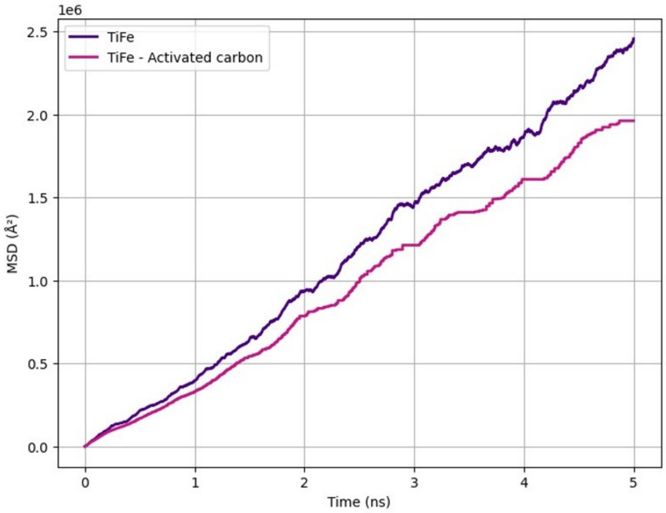

Fig. S11 | Mean square displacement (MSD) of hydrogen atoms calculated for alloy region at TiFe and TiFe–activated carbon under the NVT ensemble during production run up to 5 ns.

In pure TiFe, hydrogen atoms immediately interact with Ti and Fe sites, causing a rapid MSD increase as they diffuse through the alloy lattice. In the carbon-modified system, hydrogen must

first traverse the porous carbon layer, where motion is partially confined through adsorption, lateral migration, or temporary trapping. This initial confinement reduces early MSD values, but once hydrogen reaches the alloy, its diffusion resembles that in pure TiFe. Thus, the main effect of the carbon layer is a delayed penetration, shifting the MSD curve downward without altering mobility inside the alloy.

References

1. Wang, H., Gao, Q. & Hu, J. High hydrogen storage capacity of porous carbons prepared by using activated carbon. *Journal of the American Chemical Society* **131,** 7016–7022 (2009).
2. Sultana, A. I. & Reza, M. T. Investigation of hydrothermal carbonization and chemical activation process conditions on hydrogen storage in loblolly pine-derived superactivated hydrochars. *International Journal of Hydrogen Energy* **47,** 26422–26434 (2022).
3. Blankenship, L. S., Balahmar, N. & Mokaya, R. Oxygen-rich microporous carbons with exceptional hydrogen storage capacity. *Nature Communications* **8,** (2017).
4. Thompson, A. P., Aktulga, H. M., Berger, R., Bolintineanu, D. S., Brown, W. M., Crozier, P. S., Veld, P. J. in 't, Kohlmeyer, A., Moore, S. G., Nguyen, T. D., Shan, R., Stevens, M. J., Tranchida, J., Trott, C. & Plimpton, S. J. LAMMPS - a flexible simulation tool for particle-based materials modeling at the atomic, meso, and continuum scales. *Computer Physics Communications* **271,** 108171 (2021).
5. Hanwell, M. D., Curtis, D. E., Lonie, D. C., Vandermeersch, T., Zurek, E. & Hutchison, G. R. Avogadro: an advanced semantic chemical editor, visualization, and analysis platform. *Journal of Cheminformatics* **4,** (2012).
6. Gu, C., Zeng, S., Peng, W., You, G., Zhao, J. & Wang, Y. Molecular dynamic simulation and experiment validation on the diffusion behavior of diffusion welded FE-TI by hot isostatic pressing process. *Materials* **16,** 5626 (2023).
7. De Cachinho Cordeiro, I. M., Yuen, A. C. Y., Chen, T. B. Y., Wang, W., Yang, W., Chan, Q. N. & Yeoh, G. H. Atomistic characterisation of graphite oxidation and thermal decomposition mechanism under isothermal and Non-Isothermal heating scheme. *Computational Materials Science* **210,** 111458 (2022).
8. Jiang, H., Cheng, X.-L., Zhang, H., Tang, Y.-J. & Wang, J. Molecular dynamic investigations of hydrogen storage efficiency of graphene sheets with the bubble structure. *Structural Chemistry* **26,** 531–537 (2014).

9. Humphrey, W., Dalke, A. & Schulten, K. VMD: Visual molecular dynamics. *Journal of Molecular Graphics* **14,** 33–38 (1996).
10. Martínez, L., Andrade, R., Birgin, E. G. & Martínez, J. M. PACKMOL: A package for building initial configurations for molecular dynamics simulations. *Journal of Computational Chemistry* **30,** 2157–2164 (2009).
